# Palliative care in chronic heart failure in the Netherlands: practices, competencies and barriers among hospital-based healthcare professionals: From the Working Group of Palliative Care of the Netherlands Society of Cardiology (NVVC)

**DOI:** 10.1007/s12471-026-02083-1

**Published:** 2026-10-06

**Authors:** Marjan S. A. Aertsen-van der Kuip, Yvonne M. J. Goërtz, D. H. Frank Gommans, Sebastiaan C. A. M. Bekkers, Kirsten Boerlage-van Dijk, Margriet W. Feenema-Aardema, Carolien M. H. B. Lucas, Marike A. de Meij, Nicole H. M. K. Uszko-Lencer, Daisy J. A. Janssen

**Affiliations:** 1 Department of Cardiology, OLVG Hospital Amsterdam, Oosterpark 9, 1091 AC Amsterdam, The Netherlands; 2 Department of Health Services Research, Care and Public Health Research Institute, Faculty of Health, Medicine and Life Sciences, Maastricht University, Maastricht, The Netherlands; 3 Department of Cardiology, Maxima Medical Centre, Veldhoven, The Netherlands; 4 Department of Cardiology, Bernhoven Hospital, Uden, The Netherlands; 5 Department of Cardiology, Elkerliek Hospital, Helmond, The Netherlands; 6 Department of Cardiology, Medical Centre, Leeuwarden, The Netherlands; 7 Department of Cardiology, Alrijne Hospital, Leiderdorp, The Netherlands; 8 Department of Palliative Care, OLVG Hospital, Amsterdam, The Netherlands; 9 Department of Cardiology, Maastricht University Medical Centre, Maastricht, The Netherlands; 10 Department of Family Medicine, Care and Public Health Research Institute, Faculty of Health, Medicine and Life Sciences, Maastricht University, Maastricht, The Netherlands; 11 Department of Research and Development, CIRO, Horn, The Netherlands

**Keywords:** Chronic heart failure, Palliative care, Advance care planning, survey

## Abstract

**Background:**

Although proven beneficial, palliative care (PC) and advance care planning (ACP) for patients with chronic heart failure (HF) are still underexposed. This study aimed to (1) explore current practices of hospital-based healthcare professionals (HCPs) regarding PC and ACP in chronic HF, (2) assess their competencies, and (3) identify barriers to integrate PC and ACP into routine chronic HF care in the Netherlands.

**Methods:**

A task force of the Netherlands Society of Cardiology developed an online survey using Qualtrics. The survey was distributed among HCPs involved in the care of patients with chronic HF by the Netherlands Society of Cardiology and the Dutch Society of Cardiovascular Nurses to contacts, and through social media channels between February and March 2022.

**Results:**

120 HCPs (cardiologists (49%), nurses and nurse practitioners (40%), and allied professionals (11%)) completed the survey. 97% of HCPs found PC and ACP for patients with chronic HF (very) important, yet only about one third providing such care regularly. While 78% reported being able to recognize PC needs, only 23% felt sufficiently skilled providing PC. Reported priorities for improvement included ACP, recognition of PC needs, interprofessional collaboration, communication with patients and significant others, and sufficient time to provide PC and ACP.

**Conclusions:**

HCPs acknowledge the importance of PC and ACP in chronic HF, but integration into routine practice remains limited. Barriers are lack of skills and time constraints. There is a need for education and appropriate organization of PC and ACP for patients with chronic HF in the Netherlands.

**Supplementary Information:**

The online version of this article (https://doi.org/10.1007/s12471-026-02083-1) contains supplementary material, which is available to authorized users.

## What’s new?

- Most hospital-based HCPs acknowledge the importance of palliative care (PC) and advance care planning (ACP) in chronic heart failure (HF), but few feel adequately equipped to provide it.
- PC is frequently initiated in the end-of-life phase.
- Knowledge gaps, limited communication skills, and time constraints are key barriers to integrating PC and ACP into routine HF care.

## Introduction

Heart failure (HF), a progressive and incurable disease, is a leading cause of mortality and hospitalizations, imposing a major burden on the healthcare system [1, 2]. Despite improvements in medical therapy, the prevalence of HF increases worldwide, and many patients and their families face an unpredictable disease trajectory, high symptom burden, poor prognosis, and reduced quality of life, equivalent to malignant disease [3, 4].

Palliative care (PC) is a holistic approach aimed at improving the quality of life of patients with life-threatening illness or serious health-related suffering and their families by addressing their physical, psychosocial, and spiritual needs [5]. For patients with chronic HF, PC combined with guideline-based HF disease management has been shown to improve quality of life, communication, and symptom control [6]. To support patients with chronic HF and their families in decision-making about appropriate treatments, symptom management, and life goals, it is essential to identify PC needs in a timely manner. This involves initiating conversations about a future care plan aligned with patients’ values and preferences, also known as advance care planning (ACP) [7, 8].

Although PC and ACP are important components of HF care and the value of timely initiation has been acknowledged [9, 10], PC and ACP are still not routinely integrated into current HF care [11]. Previous studies have shown insufficient knowledge of PC and ACP and limited competencies among hospital-based healthcare professionals (HCPs), lack of training, and barriers to timely initiating appropriate PC, including the misconception that PC is solely end-of-life care. Additional barriers identified in previous studies includelimited awareness that HF is a progressive and fatal disease, prognostic uncertainty, limited interprofessional and transmural collaboration, and reimbursement challenges are barriers that have been identified in prior studies [12–14, 19–23]. While international evidence exists on current practices, barriers, and facilitators for implementing PC and ACP in chronic HF, data from the Netherlands are limited. Given that healthcare organization and implementation of PC vary across countries, insight into the Dutch context is needed. The objective of this study was threefold: (1) to explore current practices of hospital-based HCPs in the Netherlands regarding PC and ACP in chronic HF, (2) to assess the competencies of HCPs in providing PC and ACP in chronic HF management, and (3) to identify barriers to integrate PC and ACP into routine chronic HF care in the Netherlands.

## Methods

### Study design and participants

This study employed a cross-sectional descriptive survey design. HCPs involved in chronic HF care at Dutch academic and non-academic hospitals, both inpatient and outpatient, were invited to complete an online survey using Qualtrics between February 1 and March 1, 2022. The survey was developed by the Task Force on PC of the Dutch professional association ‘Netherlands Society of Cardiology’ (Dutch: Nederlandse Vereniging voor Cardiologie (NVVC)). Their objectives included identifying barriers to ACP and PC in HF, changing the culture and perceptions of ACP and PC by means of education and training, increasing awareness of PC among HCPs, patients, and relatives, and enhancing transmural PC. The Task Force consisted of five cardiologists, a cardiologist in training, a general practitioner (GP), an elderly care physician/PC physician, and a nurse practitioner, all experts in PC and ACP for patients with HF.

Medical ethics approval was not required because this online survey does not fall under the Medical Research Involving Human Subjects Act. Participants were asked to provide consent to use their response options for research purposes.

### Data collection procedure

The electronic survey link, using Qualtrics, and accompanying study information were distributed to HCPs by the NVVC by newsletter (~1900 members) and the Dutch Society of Cardiovascular Nurses by social media, emails, and a newsletter (~4200 contacts) between February and March 2022. The survey consisted of 28 questions and included items regarding demographical characteristics (e.g., age, gender, profession, and years in practice) of the HCPs, their role in PC and ACP, knowledge and skills regarding PC and ACP, education, collaboration agreements with GPs and other involved HCPs, and personal needs for training in ACP and PC.

### Statistical analysis

The online survey responses were analysed using descriptive statistics. Data were presented as means ± standard deviations (SDs), or frequencies and proportions, as appropriate. Data analysis was performed using IBM SPSS Statistics 28.0.1.1. (SPSS, Inc., Chicago, IL, USA). Open-ended responses were analysed using a simple thematic approach. Responses were reviewed and grouped into recurring themes based on their content. This process was performed independently by two researchers (MSAA and YMJG), and findings were discussed until consensus was reached.

## Results

### Demographics

In total, 121 HCPs completed the survey. One HCP did not provide consent for the use of their data for research purposes. Therefore, 120 HCPs participated in the present study, including 62 cardiologists, 48 nurses and nurse practitioners, and 10 allied health professionals. Most of the HCPs were female (72%), and the mean age was 45 years. Work experience ranged from 1 to 45 years (Tab. 1).

**Table 1:** Health care professionals’ characteristics (*n* = 120)

Female, *n* (%)	86 (72)
Age (years), mean ± SD	45.2 ± 10.5
*Profession*	
Cardiologist, *n* (%)	62 (52)
Nurse and nurse practitioner, *n* (%)	48 (40)
Allied professional, *n* (%)	10 (8)
Years in function working in the current profession, mean ± SD [min-max]	13.0 ± 10.5 [1.0–45.0]

*n* number*, SD* standard deviation,* min* minimum, *max* maximum

### Current practices

Nearly all HCPs (97%) considered providing PC and ACP for patients with HF to be (very) important. The majority of HCPs (78%) indicated that PC should be offered when symptoms worsen despite optimal HF treatment. Other commonly reported times points for initiating PC included at the time of HF diagnosis (16%), during the end-of-life phase (31%), when advanced therapies (e.g. implantable cardioverter-defibrillator, cardiac resynchronization therapy, left ventricular assist device, heart transplantation) are being considered (37%) and recurrent hospitalizations for HF (62%). Respondents were allowed to select multiple answers, the majority (62%) chose multiple options.

Most respondents (65%) (strongly) agreed that every cardiologist (in training) in the Netherlands should be able to provide PC, whereas a smaller proportion (23%) was neutral and 12% (strongly) disagreed. About half (51%) of the cardiologists responded that within their team a cardiologist was actively involved in PC, either themselves (8%), in collaboration with other cardiologists within their team (11%) or through a colleague (32%).

When asked about the preferred structure for delivering PC, 75% of respondents indicated that it should primarily be delivered through shared care, involving collaboration among cardiologist, nurse practitioner, PC team, and GP. Regarding consultation practices, 81% of HCPs reported consulting the PC team for PC or ACP-related questions, while other HCPs reported consulting the cardiologist (33%), nurse practitioners/physician assistant (20%), or GP (17%); 4% selected “other”. Half of the respondents consulted only one discipline, while the other half reported consulting multiple sources. Cardiologists (84%) stated they seek guidance from the PC team for patients with HF, and 39% indicated that a cardiologist or another HCP from the cardiology department was formally a member of the PC team.

### Frequency of PC and ACP provision

Sixty-seven percent of HCPs indicated providing PC 1 to 10 patients per year, while 8% reported not providing PC at all (Tab. 2). Nearly half of HCPs reported that their cardiology practice provides PC and ACP occasionally, one third regularly and one fifth seldom or never. On average, HCPs reported conducting 1 to10 ACP conversations each year, with 29% reporting that they did not conduct any ACP conversations (Tab. 3).

**Table 2:** Number of patients to whom HCPs provide palliative care per year (*n* = 120)

0	10 (8%)
1–10	81 (67%)
11–25	19 (16%)
26–50	8 (7%)
> 51	2 (2%)

Presented as number and percentage

*HCP* healthcare professional

**Table 3:** Number of advance care planning conversations per year by HCPs (*n* = 120)

0	35 (29%)
1–10	47 (39%)
11–25	28 (23%)
26–50	7 (6%)
> 51	3 (3%)

Presented as number and percentage

*HCP* healthcare professional

### Competencies, education & training

**Fig. 1:**
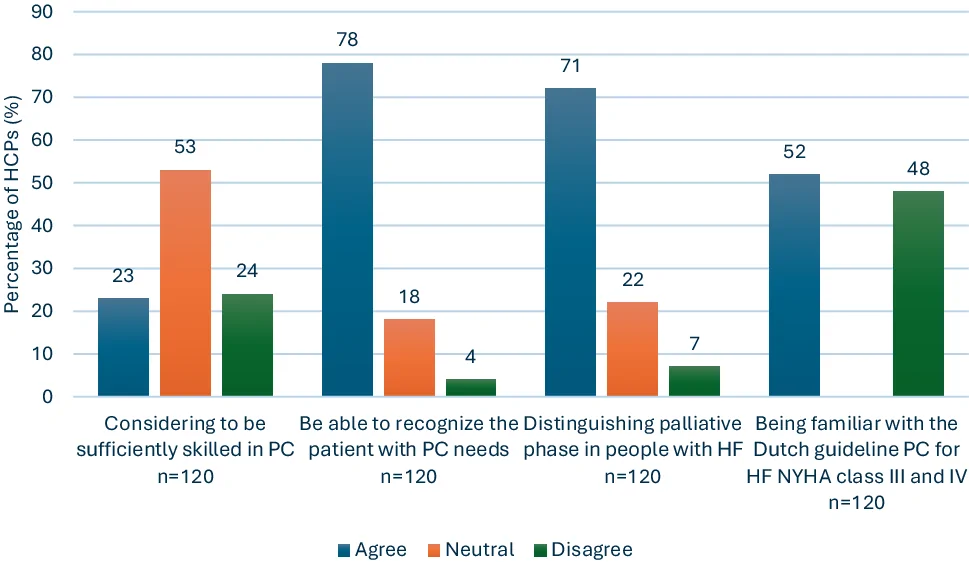
Healthcare professionals’ competencies in palliative care. *HCP* healthcare professional,* PC* palliative care, *HF* heart failure,* NYHA* New York Heart Association

The majority of HCPs (78%) reported being able to recognize PC needs, whereas 23% considered themselves sufficiently skilled to provide PC. Most HCPs (71%) reported being able to identify the palliative phase in HF. In addition, half of the HCPs (52%) reported that they were familiar with the Dutch guideline ‘Palliative care in heart failure NYHA class III-IV’ ([16]; Fig. 1). Of these, 47% of HCPs indicated that they applied this guideline in daily practice. Moreover, 38% of HCPs were aware of tools available to identify PC needs, such as ‘Supportive and Palliative Care Indicators’ (SPICT) [17] and the Dutch tool ‘Identification of patients with HeARt failure with Palliative care needs’ (I-HARP) [18]. The majority of HCPs (81%) indicated they would benefit from more training in ACP and PC. About 35% of the HCPs had completed additional training in PC such as a 9-day PC training for medical specialists, a 1-year post-bachelor training for nurses, a minor during bachelor training for nurses or internships.

### Barriers

**Fig. 2:**
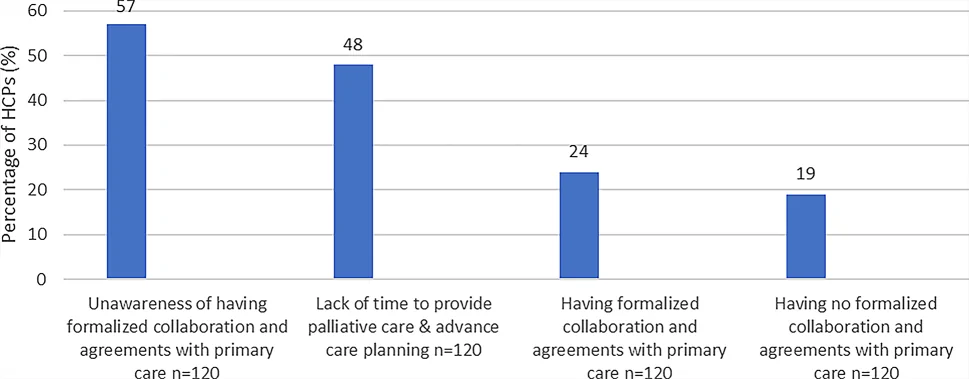
Barriers in palliative care and advance care planning. *HCP* healthcare professional

**Fig. 3:**
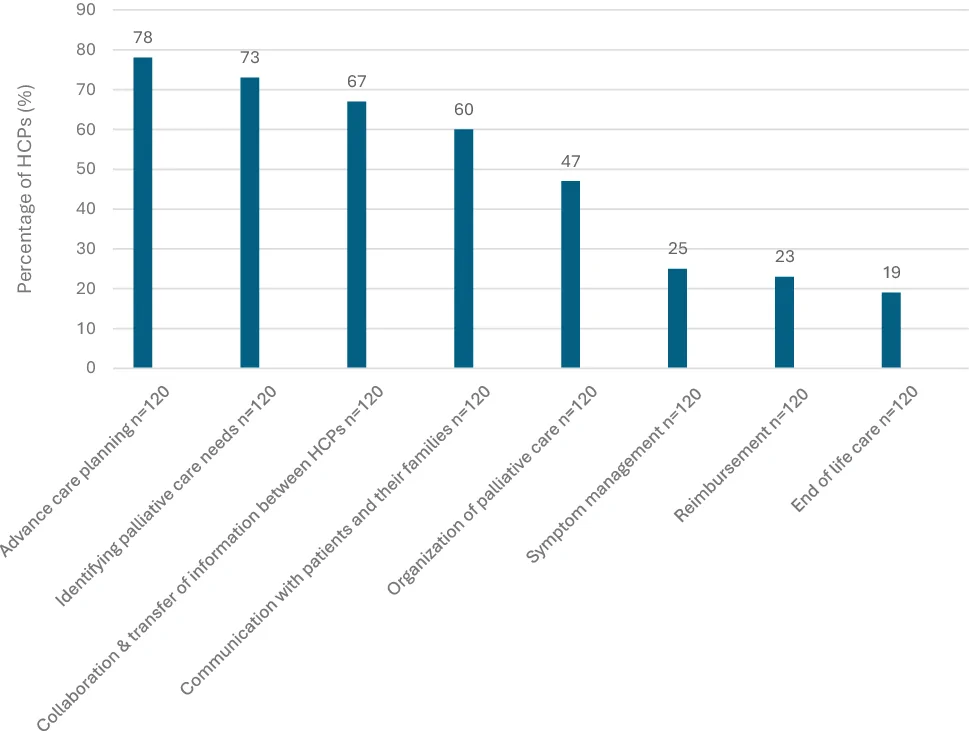
Areas regarding improvement in palliative care. *HCP* healthcare professional

Almost half of the HCPs (48%) indicated that time constraints were a barrier to providing PC and ACP. Formalized collaboration and agreements with primary care were limited: 24% indicated having agreements in place, 57% were unaware of any such agreements, and 19% stated their practice had no formal collaboration agreements. The vast majority (92%) indicated that two or more barriers of PC delivery required improvement (Fig. 2). Regarding areas for improvement in PC, HCPs most frequently identified ACP (78%), followed by identifying PC needs (73%), collaboration and information transfer between HCPs (67%), communication with patients and their families (60%), and organization of PC (47%). Symptom management (25%), reimbursement (23%), and end-of-life care (19%) were less frequently mentioned, though still noted as areas for improvement (Fig. 3).

## Discussion

This study provides insight into current practices, competencies, and perceived barriers regarding the integration of PC and ACP into hospital-based HF care in the Netherlands. While most hospital-based HCPs recognize the importance of PC and ACP and most consult the PC team for ACP- or PC-related questions, only one-third of HCPs routinely provide such care or feel adequately prepared to do so. Although most HCPs felt able to recognize patients with PC needs, far fewer considered themselves sufficiently skilled to conduct these conversations, and knowledge of guidelines and tools was limited. About one third of HCPs reported the end-of-life phase as the moment to initiate PC. The findings point to key areas for improvement: knowledge of PC and ACP, communication skills and providing PC and ACP, awareness of guidelines and tools for identifying PC needs, and addressing structural barriers such as time constraints.

Regarding current practices, our study shows that PC and ACP are still not routinely embedded in Dutch hospital-based HF care, despite recommendations in guidelines [3, 15, 18].

Although most respondents acknowledged the importance of PC and ACP, only about one-third reported providing PC or ACP on a regular basis, while 8% reported never providing PC and 29% reported never engaging in ACP. This discrepancy between recognition and actual practice is in line with previous studies [21–23]. In addition to the limited frequency of PC and ACP provision, one-third of respondents indicated the end-of-life phase as appropriate timeto initiate PC conversations. This aligns with earlier studies reporting that PC in HF is often introduced late in the disease trajectory [11, 13]. While this reflects current practice, it raises questions about the timing of initiation in the disease trajectory.

Concerning competencies, respondents indicated that they often lack knowledge and training in PC, as well as communication skills, particularly when addressing conversations with patients and families about future care plans, values, preferences, or end-of-life care. Only a minority felt sufficiently skilled to engage in these conversations. This aligns with previous research [7, 19, 20] and underscores the need for training programs that include both knowledge and communication skills.

Beyond individual-level barriers, respondents also identified several structural and organizational factors that may limit the integration of PC and ACP into current practice. Time constraints, limited collaboration or the absence of formal collaboration agreements were also reported as important barriers. These findings are consistent with previous studies [12, 14, 20] and highlight that, in the Dutch hospital context, education of HCPs alone will not be sufficient. Organizational support, policy measures, and reimbursement are equally required to enable consistent and timely integration of PC and ACP into routine HF care. Recent policy changes in the Netherlands may facilitate such integration. Since January 2025, reimbursement for ACP consultations has been available, with the requirement that the care plan is shared transmurally with all relevant HCPs, including the GP [24]. Ensuring these policies are matched by adequate PC and ACP training will be the key to successful implementation in practice.

### Strengths and limitations

This study is to our knowledge the first to investigate current practices, competencies, and barriers regardingPC and ACP among hospital-based HCPs in the Netherlands. It provides valuable insights into the training and support needs of HCPs involved in HF care in Dutch hospitals. As healthcare systems differ internationally, the applicability of these findings to other countries may be limited. Several limitations of this study must be considered. First, this study focused solely on hospital-based HCPs. Therefore, the results cannot be directly translated to primary or tertiary care. Another limitation of this study is the underrepresentation of male respondents, occupational therapists, physician assistants, and GPs which may have influenced our findings. The characteristics of the study population, including the proportion of participants with additional training in PC, may reflect a group with particular interest in PC. This may have influenced the findings and could have resulted in an overestimation of real-world practice. Further, because of the survey distribution strategy used, the response rate cannot be determined. Finally, because data were self-reported, they may reflect perceived rather than actual practices and competencies.

## Conclusions and implications

Hospital-based HCPs in the Netherlands, consider PC and ACP as important in chronic HF care, but PC and ACP are not yet routinely embedded in daily practice. This study highlights a clear need to improve knowledge and training focused on PC and ACP needs, as well as enhanced skills in communication about PC and ACP.

Training in PC and ACP should be integrated into the educational programs of HCPs involved in HF care. The study also shows that appropriate organization, sufficient time for PC and ACP conversations, financial reimbursement, and formal interprofessional and transmural collaboration agreements are needed for further improvement of PC and ACP for patients with chronic HF.

## Supplementary Information

Survey ‘Palliative care in chronic heart failure in the Netherlands: practices, competences and barriers among hospital-based healthcare professionals’

## Data Availability

The data supporting the findings of this study are available from the corresponding author upon reasonable request.
