# Supplementary material for: Palliative care in chronic heart failure in the Netherlands: practices, competencies and barriers among hospital-based healthcare professionals: From the Working Group of Palliative Care of the Netherlands Society of Cardiology (NVVC)
Source: Neth Heart J. 2026 Oct 6;34(11):414–9. doi: 10.1007/s12471-026-02083-1 (PMC13694642; doi:10.1007/s12471-026-02083-1)
Supplement: Supplementary file 1 [file 12471_2026_2083_MOESM1_ESM.docx]

**Survey ‘Palliative care in chronic heart failure in the Netherlands: practices, competences and barriers among hospital-based healthcare professionals’**

**Netherlands Society of Cardiology (NVVC) - Working Group of Palliative Care**

1.       What is your gender?

2.       What is your profession?

3.       Is a cardiologist in your team actively involved in palliative care?

4.       Have you completed any additional training in palliative care during or after your professional education/specialization?

5.       I consider myself sufficiently competent in the field of palliative care provision

6.       I know the Dutch guideline ‘Palliative care in heart failure NYHA class III and IV’ Version 3 (2018)

7.       I apply the Dutch guideline ‘Palliative care in heart failure NYHA class III and IV’ Version 3 (2018) in my patient care

8.       I consider the provision of palliative care and advance care planning to patients with a chronic life-threatening disease such as heart failure, as…

9.       I am able to identify the patient with palliative care needs

10. I am able to distinguish a palliative phase in patients with heart failure

11.    Are you aware of the different instruments available for initiating palliative care, such SPICT and I-HARP?

12.    When should palliative care be initiated during the clinical course of chronic heart failure? (multiple answers possible)

13.    Do you know whom to consult when having questions concerning palliative care or advance care planning?

14.    Whom do you consult when you have questions regarding palliative care and advance care planning for one of your patients? (multiple answers possible)

15.    How frequently does your cardiology practice provide palliative are and/or advance care planning for patients with chronic cardiac disease, such as heart failure?

16.    To how many patients do you personally provide palliative care per year?

17.    With how many patients do you conduct advance care planning discussions per year?

18.    Every cardiologist (including those in training) in the Netherlands should be capable of providing palliative care themselves

19.    Which health care professional do you believe should primarily deliver palliative care to patients with chronic cardiac disease? (multiple answers possible)

20.    Does your hospital have a palliative care-team?

21.    Is the palliative care-team in your hospital consulted by cardiologists of for patients with cardiac disease?

22.    Is the cardiology department (including cardiologists or other health care providers involved in care for patients with cardiac disease) actively involved in the palliative care-team?

23.    The palliative care-team in my hospital possesses sufficient expertise regarding patients with chronic cardiac disease

24.    I have sufficient time to provide palliative care and conduct advance care planning

25.    Does your cardiology practice of region have a formalized collaboration with primary care, included agreements regarding primary care?

26.    Which aspects of palliative care for patients with heart failure require improvement? (multiple answers possible)

27.  I would like to receive additional education regarding advance care planning and palliative care provision

28.    What do you require in your practice (inpatient and/or outpatient) to provide palliative care? (open-ended question).
